# Rapidly Evolving Landscape and Future Horizons in Hepatocellular Carcinoma in the Era of Immuno-Oncology

**DOI:** 10.3389/fonc.2022.821903

**Published:** 2022-03-31

**Authors:** Sandra Mirie Kang, Lana Khalil, Bassel F. El-Rayes, Mehmet Akce

**Affiliations:** Department of Hematology and Medical Oncology, Winship Cancer Institute of Emory University, Atlanta, GA, United States

**Keywords:** hepatocellular carcinoma, immunotherapy, tumor microenvironment, immune checkpoint inhibitor, molecular targeted agents

## Abstract

Hepatocellular carcinoma (HCC) is a serious global health problem as one of the leading causes of cancer-related death worldwide. Systemic therapy for advanced HCC has progressed with the development of molecular targeted agents, however survival benefits remain modest. More recently, immune checkpoint inhibitors (ICI) have emerged and exhibited promising therapeutic benefits in a subset of patients. Physiologically, the intrinsic microenvironment in the liver is immunosuppressive, which represents a major obstacle for effective immune therapies in primary and secondary liver malignancies. For this reason, combination therapies that can overcome immune inhibitory mechanisms and enhance the immune response are a rationale approach for drug development in HCC. A recent example is the combination of the anti-PD-L1 antibody (atezolizumab) and anti-VEGF-A antibody (bevacizumab), which has shown significant improvement in survival as compared to standard of care in the first-line treatment for HCC. Other immunotherapy approaches including cancer vaccines and adoptive cell therapy are also under investigation. This review summarizes the key trials leading to our current HCC treatment options and provides an overview of future immune-based strategies in development.

## Introduction

Hepatocellular carcinoma (HCC) is the sixth most common cancer and fourth leading cause of cancer mortality worldwide with approximately 800,000 deaths per year. Although the incidence rates of most malignancies are declining, the incidence rate of HCC continues to increase. It is estimated that over one million individuals will develop HCC annually by 2025 (1, 2). The majority of HCC cases (>70%) occur in Asia, however numbers in the Western world are rising. Chronic liver disease due to Hepatitis B (HBV) and C (HCV) viruses are the most common causes of HCC, followed by other etiologies, including excess alcohol intake, non-alcoholic fatty liver disease (NAFLD) associated with metabolic syndrome, and environmental toxins (2, 3). The prognosis of HCC is largely determined by the stage at diagnosis. Potentially curative treatment options including surgical resection and liver transplantation are offered with earlier stages of disease and result in over 70% 5-year survival rates (4). Unfortunately, the majority of patients present with advanced stage HCC, which has dismal long-term survival rates. Clinical challenges in the management of advanced stage HCC include the underlying medical liver disease, altered liver functions, and systemic effects of liver dysfunction which can complicate side effects of commonly used therapies (4, 5).

Prior to the advent of sorafenib, systemic therapy for advanced stage HCC was limited to cytotoxic agents (doxorubicin), which historically have shown poor response rates (< 25%) and significant toxicities (6). Over the past decade, there has been significant advancement in HCC treatment with the development of molecular targeted agents and immune therapies. Sorafenib was the first multi-targeted tyrosine kinase inhibitor (TKI) to demonstrate a survival benefit in advanced HCC. Later studies demonstrated clinical benefits from other TKIs, including lenvatinib (7), cabozantinib (8), and regorafenib (9). Although the advent of TKIs was a major breakthrough in HCC treatment, the prognosis remained poor with a median overall survival of 10-14 months, highlighting the unmet need to develop novel therapies to further improve patient survival outcomes (7–9).

In recent years, immunotherapies have rapidly changed the scope of cancer treatment with the growing recognition that immune evasion is an important mechanism of cancer progression (10). The effectiveness of immunotherapy demonstrated in other cancers like melanoma led to studies evaluating its use in HCC (11). Immune checkpoint inhibitors (ICI), including atezolizumab combined with bevacizumab, pembrolizumab, and nivolumab combined with ipilimumab, have since been approved for treatment of advanced HCC and are now incorporated into current HCC treatment guidelines (12–15). In this review, we will provide an update about the current landscape of the systemic therapies in advanced HCC (**Table 1**) and discuss novel strategies on the horizon in the era of immuno-oncology.

**Table 1:** Key findings of landmark clinical trials for the approved systemic therapies in advanced HCC.

Trial Name	Treatment Arms	Line of Therapy	Primary Endpoint	ORR (%)	PFS (months)	OS (months)	HR	Approval Date
SHARP (7)	Sorafenib vs. placebo	First	OS	2 vs. 1	5.5 vs. 2.8	10.7 vs. 7.9	0.69	2007
RESORCE (9)	Regorafenib vs. placebo	Second	OS	11 vs. 4	3.1 vs. 1.5	10.6 vs. 7.8	0.63	2017
REFLECT (8)	Lenvatinib vs. Sorafenib	First	OS	24.1 vs. 9.2	7.4 vs. 3.7	13.6 vs. 12.3	0.92	2018
CELESTIAL (16)	Cabozantinib vs. placebo	Second and Third	OS	4 vs. 1	5.2 vs. 1.9	10.2 vs. 8	0.76	2019
REACH-2 (17)	Ramucirumab vs. placebo (AFP > 400 ng/mL)	Second	OS	5 vs. 1	2.8 vs. 1.6	8.5 vs. 7.3	0.71	2019
CheckMate-040 (18)	Nivolumab	Second	ORR	15	3.4 (dose-escalation)	15	N/A	2017
KEYNOTE-224 (13)	Pembrolizumab	Second	ORR	17	N/A	N/A	N/A	2018
CheckMate-040 (14)	Nivolumab + Ipilimumab	Second	ORR	32	N/A	N/A	N/A	2020
IMbrave150 (12)	Atezolizumab + Bevacizumab vs. Sorafenib	First	OS, PFS	29.8 vs. 11.3	6.8 vs. 4.3	19.2 vs. 13.4	0.66	2020

N/A, Not applicable.

## Liver Tolerogenicity and HCC Immune Evasion

The liver has a unique immunosuppressive microenvironment that prevents overactivation of the immune system from constant exposure to antigens arising from the gut (19). Liver sinusoidal endothelial cells (LSECs) form the fenestrated barrier between the liver parenchyma and sinusoids, and act as antigen-presenting cells (APCs). However, LSECs express high levels of immunosuppressive receptors like program death receptor ligand 1 (PD-L1) and low levels of costimulatory molecules CD80 and CD86, decreasing their ability to activate T cells. This is largely due to their role of limiting immune responses to gut bacterial molecules in order to avoid unnecessary inflammatory tissue damage (19, 20). Kupffer cells (KCs) are the liver-residing macrophages with the primary function of pathogen clearance (21). Similar to LSECs, KCs induce tolerance by low expression of major histocompatibility complex (MHC) molecules, secretion of immunosuppressive cytokines like IL-10 and prostaglandins, and direct activation of inhibitory forkhead box P3 (FoxP3) that leads to expansion of regulatory T cells (Tregs) (22). Hepatic dendritic cells (DCs) also express low levels of co-stimulatory molecules and MHC as well as produce anti-inflammatory prostaglandins (23). Overall, this immunosuppressive microenvironment is necessary to maintain self-tolerance in the liver, although it poses a challenge to the development of anti-tumor immunity in HCC.

HCC development and progression are typically characterized by chronic inflammation of the liver caused by infection, toxins, and steatosis (24). Within this inflammatory state, multiple complex immunosuppressive mechanisms become activated in HCC and contribute to immune evasion. In HCC, immunosuppressive cytokines like IL-10 and TGF-β are continuously expressed and immune cells including Tregs, myeloid-derived suppressor cells (MDSCs), and M2 tumor-associated macrophages (TAM), accumulate in the liver (25, 26). MDSCs are immature myeloid cells that inhibit effector T cell function, increase expansion of Tregs, and upregulate PD-L1 (27). The increased interaction between programmed cell death protein (PD-1) on T cells and PD-L1 on cancer cells promotes T cell exhaustion or dysregulation, and higher numbers of PD-1+ CD8+ T cells have been associated with disease progression and poorer prognosis (28–30). Defects in APC molecules also occur in HCC, resulting in downregulation of HLA-1 expression and thus ineffective presentation of tumor antigens that allows for escape from cytotoxic T cells (31). Together, these mechanisms represent potential treatment strategies in order to enhance anti-tumor immunity in HCC, some of which have been successfully utilized to develop FDA-approved agents and others that are currently under investigation.

## Current Treatment Options

### Molecular Targeted Agents

#### Sorafenib

Sorafenib inhibits the activity of multiple tyrosine kinases involved in tumor angiogenesis and proliferation, including vascular endothelial growth factor receptors (VEGFR) 1-3, Raf kinases, and platelet-derived growth factor receptor beta (PDGFR-β). The landmark phase III SHARP trial randomized 602 previously untreated patients, predominantly from a Western population, with advanced HCC to receive either sorafenib (400 mg twice daily) or placebo. Median overall survival (OS) was significantly better in the sorafenib arm at 10.7 months versus 7.9 months in the placebo group (Hazard ratio (HR) 0.69, p<0.001) (7). Similar positive results were reported in a parallel Asia-Pacific cohort (median OS 6.5 months for sorafenib vs. 4.2 months for placebo; HR 0.68, p=0.014). A potential explanation for the survival differences between the trials is the higher risk baseline tumor and patient characteristics in the Asia-Pacific study, including more patients with extrahepatic spread, higher alpha-fetoprotein (AFP) levels, and poorer ECOG performance status. Both studies notably restricted enrollment to patients with Child-Pugh A disease and reported similar treatment-related adverse events including hand-foot syndrome and diarrhea (7, 32). The GIDEON study observed over 3,000 HCC patients treated with sorafenib and found comparable safety profiles among Child-Pugh class A and B patients, suggesting that treatment may be used in higher degrees of liver dysfunction. However, the results should be interpreted with caution given the observational nature of the study, and treatment should be carefully assessed in those with more clinically significant underlying liver disease (33).

#### Regorafenib

Regorafenib inhibits multiple TKIs, including VEGFR 1-3, KIT, PDGFR-β, fibroblast growth factor receptors (FGFR) 1-2, BRAF and RET. The phase III RESORCE trial randomized 573 patients who progressed on sorafenib to either regorafenib (160 mg once daily for three weeks on and one week off) or placebo. Regorafenib resulted in significant prolongation in median OS (10.6 vs. 7.8 months, HR 0.63, p<0.0001) as well as median time to progression (TTP) (3.2 vs. 1.5 months) and objective response rates (ORR) (11% vs. 4%). Side effects of regorafenib were similar to those reported for sorafenib, including hand-foot syndrome, hypertension, and fatigue (9). Nearly one decade after sorafenib approval, regorafenib was the next TKI approved as second-line therapy for HCC (34).

#### Lenvatinib

Following sorafenib approval, multiple randomized phase III trials evaluating other drugs (sunitinib, brivanib, linifanib, erlotinib plus sorafenib) for first-line treatment failed (35–39). Lenvatinib is a multikinase inhibitor with activity against VEGFR 1-3, PDGFR-α, FGFR 1-4, KIT and RET. Based on promising results from a phase II study, the phase III non-inferiority REFLECT trial randomized 954 patients with unresectable HCC and Child-Pugh A disease to lenvatinib (12 mg/day for bodyweight ≥60 kg or 8 mg/day for bodyweight <60kg) or sorafenib (400 mg twice daily) in the first-line setting (8, 40). Lenvatinib was noninferior to sorafenib for the primary endpoint of median OS (13.6 vs. 12.3 months; HR 0.92). Lenvatinib also led to significantly higher progression-free survival (PFS) (7.4 vs. 3.7 months; HR 0.66, p<0.0001), median TTP (8.9 vs. 3.7 months; HR 0.63, p<0.0001), and ORR (24.1% vs. 9.2%; odds ratio (OR) 3.13, p<0.0001) compared to the control arm. Hypertension was more common for lenvatinib and hand-foot skin reaction for sorafenib, but toxicity profiles were overall relatively similar (8). Moreover, an exploratory analysis of the REFLECT trial demonstrated that objective response was an independent predictor of OS in patients with advanced HCC (median OS 22.4 months for responders vs. 11.4 months for nonresponders) (41).

#### Cabozantinib

The effectiveness of cabozantinib, a potent inhibitor of VEGFR1-3, MET, and AXL, in previously treated advanced HCC was shown in the phase III CELESTIAL trial. The study enrolled 707 patients with advanced HCC with up to two prior lines of treatment and assigned them to either cabozantinib (60 mg daily) or placebo. Cabozantinib achieved superior survival outcomes compared to placebo, with median OS 10.2 months versus 8 months (HR 0.76, p=0.005), median PFS 5.2 months versus 1.9 months (HR 0.44, p<0.001), and ORR 4% versus <1% (16). Survival benefit with cabozantinib was seen across high (≥400 ng/mL) and low (<400 ng/mL) baseline AFP levels, and resulted in more AFP responses (≥20% decrease from baseline at Week 8) that was associated with longer OS and PFS (42). Side effects of cabozantinib were similar to those of sorafenib, including hand-foot syndrome and hypertension (16).

#### Ramucirumab

Ramucirumab is a recombinant IgG1 monoclonal antibody (mAb) that inhibits VEGFR-2. A phase II study of 42 patients showed promising results of ramucirumab in the first-line setting, but did not make direct comparisons with sorafenib (43). Subsequently, the phase III REACH trial randomized 565 HCC patients after progression on sorafenib to ramucirumab (8 mg/kg every 2 weeks) or placebo, which showed no significant differences in median OS (9.2 months for ramucirumab vs. 7.6 months placebo, HR 0.87, p=0.14). However, a subgroup analysis indicated a significant survival benefit for patients with elevated AFP levels of >400 ng/mL (median OS for ramucirumab 7.8 months vs. 4.2 months placebo; HR 0.67, p=0.006) (44). The benefits seen in this particular subset of patients were validated by the REACH-2 study, which demonstrated an improved median OS of 8.5 months with ramucirumab compared to 7.3 months in the placebo arm (HR 0.71, p=0.019), and PFS was 2.8 months vs. 1.6 months (HR 0.45, p<0.0001) (17, 44). These findings could potentially be explained by the association between elevated AFP and increased angiogenesis, resulting in increased sensitivity to VEGFR-2 inhibition. Ramucirumab was generally well-tolerated, with peripheral edema and ascites reported as the most frequent treatment-related adverse events, and has the advantage of no associated hand-foot skin reaction (44).

### Immunotherapy

#### Nivolumab

Nivolumab, a human monoclonal antibody that targets PD-1, was the first ICI approved for HCC (45). CheckMate-040 was a phase I/II dose-escalation and expansion study that overall demonstrated safety and efficacy of nivolumab in advanced HCC. Patients with Child-Pugh B7 disease, viral hepatitis (HBV or HCV), and previous sorafenib treatment were also included. In the dose-escalation phase, patients received 0.1 to 10 mg/kg of nivolumab every 2 weeks while those in the expansion phase were treated with 3 mg/kg every 2 weeks. Objective response rates were between 15-20% with median durations of response ranging from 9.9 to 17 months across both phases of the study. The median OS was 15 months in the dose-escalation phase, and even patients who had progressed on sorafenib achieved a median OS of 13.2 months. Most adverse events were manageable and comparable between those with Child-Pugh A and B disease (18, 46). These results led to the accelerated approval of nivolumab for second-line HCC treatment by the FDA in 2017 (45). This was later followed by the phase III CheckMate-459 trial that randomized 743 patients with advanced HCC to either nivolumab or sorafenib in the first-line setting. Although the primary endpoint of OS did not meet the predefined threshold of statistical significance (HR 0.83, p=0.0419), nivolumab showed a trend towards improved median OS (16.4 vs. 14.7 months; HR 0.85, p=0.0752) and ORR (15% vs. 7%) compared to sorafenib. Patients in the nivolumab arm also had fewer grade 3/4 treatment-related adverse events (22% vs. 49%) and lower rates of discontinuation (4% vs. 8%) (47). Based on these findings, the FDA did eventually withdraw accelerated approval of nivolumab, however it could be a potential option particularly for patients ineligible for TKIs or other anti-angiogenic agents and in those with Child-Pugh B per NCCN guidelines (15, 48).

#### Pembrolizumab

KEYNOTE-224 was a phase II trial that showed efficacy and tolerability of pembrolizumab in HCC previously treated with sorafenib (ORR 17%, 44% with stable disease), leading to FDA accelerated approval of the anti-PD-1 antibody as a second-line therapy option in 2018 (13, 49). Similar response rate was reported in the subsequent KEYNOTE-240, a phase III randomized control trial that assigned 413 patients on second-line treatment to best supportive care with or without pembrolizumab (200 mg every 3 weeks). Although pembrolizumab did not improve median OS (13.9 vs. 10.6 months, HR 0.78) and PFS (3 vs. 2.8 months) compared to placebo, ORR was significantly better (18.3% vs. 4.4%) with a considerable median duration of response of 13.8 months. The safety profile of pembrolizumab was manageable; grade 3 or higher immune-related adverse events occurred in 7.2% of patients and no flares of viral hepatitis were reported (50).

### Combination Immune therapy

#### Nivolumab/Ipilimumab

One cohort of CheckMate-040 assessed the combination of nivolumab plus ipilimumab (anti-cytotoxic T-lymphocyte antigen-4 [CTLA-4]) in 148 cases of sorafenib-treated HCC. Patients were randomized to one of three arms composed of different doses and schedules of nivolumab plus ipilimumab. Across the three groups, ORR was 31% and eight patients had complete responses. Forty-nine patients in one arm received nivolumab 1 mg/kg plus ipilimumab 3 mg/kg every 3 weeks for 4 doses followed by nivolumab 240 mg every 2 weeks until disease progression or intolerability (14). This arm compared to the others had the best median OS at 22.8 months (vs. 12.5 and 12.7 months in the other groups) and disease control rate of 54% (vs. 43% and 49% in the other groups) (50). Responses were observed regardless of baseline PD-L1 status (14). The combination of nivolumab plus ipilimumab was soon approved by the FDA in 2020 for HCC after previous sorafenib treatment (51).

#### Atezolizumab/Bevacizumab

Bevacizumab, an anti-VEGF-A monoclonal antibody, has been evaluated as monotherapy and in combination with various cytotoxic agents for advanced HCC in multiple phase II studies (52–54). The landmark IMbrave150 trial demonstrated superiority of bevacizumab plus atezolizumab (anti-PD-L1) over sorafenib (12, 55). Prior to this trial, a phase 1b study had shown promising results from the combination, with an ORR of 36% and median PFS of 5.6 months while maintaining an acceptable toxicity profile (56). The IMbrave150 trial randomly assigned 501 patients to either atezolizumab plus bevacizumab (1200 mg and 15 mg/kg, respectively, every 3 weeks) or sorafenib (400 mg twice daily). This treatment combination compared to standard care significantly improved survival outcomes (HR 0.58, p<0.001) with higher 1-year OS rates (67.2% vs. 54.6%) and median PFS (6.8 vs. 4.3 months) (12). An updated analysis showed that survival benefit with the combination was sustained; median OS with atezolizumab plus bevacizumab was 19.2 months versus 13.4 months with sorafenib (HR 0.66, p=0.0009). The frequency of grade 3/4 adverse events was similar between both arms, with hypertension and proteinuria as the most common side effects of the combination treatment (12, 57). Importantly, analyses of patient-reported outcomes showed clinically meaningful improvements in reported quality of life, disease symptoms, and functioning with atezolizumab plus bevacizumab over sorafenib (58). The approval of this combination marked a significant shift in HCC treatment, with an immunotherapy-based regimen now recommended as the preferred first-line systemic therapy option based on improved survival (15).

## Future Options

### ICI Plus ICI Combinations

Promising results from ICI therapies in recent clinical trials have led to the investigation of other ICI-based combinations and immunotherapy strategies in advanced HCC (**Table 2**). The combination of different checkpoint inhibitors is appealing due to its potential to overcome resistance to single-agent immunotherapy by targeting multiple pathways the tumor uses to evade the immune response (28). After nivolumab plus ipilimumab demonstrated efficacy in sorafenib progressors, the anti-PD-1/CTLA-4 combination is currently being compared to sorafenib and cabozantinib as first-line treatment in CheckMate-9DW (NCT04039607) (14, 60). Durvalumab (anti-PD-L1) plus tremelimumab (anti-CTLA-4) is a similar ICI combination that showed significant clinical activity and tolerability for unresectable HCC after sorafenib in a phase I/II study (76). Recent presentation of the phase III HIMALAYA trial (NCT03298451) evaluating the STRIDE (Single T [tremelimumab] Regular Interval D [durvalumab]) regimen versus sorafenib demonstrated superior median OS (16.4 vs. 13.8 months, HR 0.78, p=0.0035) and ORR (20.1% vs. 5.1%) with comparable safety profiles. Additionally, single agent durvalumab was noninferior to sorafenib, with a median OS of 16.6 versus 13.8 months (HR 0.86, noninferiority margin 1.08) and ORR of 17%. Based on these results, dual ICI combination and single agent durvalumab may become potential first-line systemic therapy options for advanced HCC in the near future (61, 77). Other potential targets of checkpoint blockade include TIM-3 and LAG-3, which are both expressed along with PD-1 on CD8+ T cells and contribute to T cell dysfunction and suppression (78). Phase II trials of dual checkpoint blockade against PD-1 and LAG-3 (NCT04567615) as well as TIM-3 (NCT03680508) in HCC are ongoing (62, 63). Safety is a notable concern in combination immunotherapy, as shown in CheckMate-040 with up to 53% of patients on nivolumab plus ipilimumab developing grade 3/4 treatment-related adverse events (e.g. immune hepatitis, pneumonitis) (14). Therefore, patients should be monitored closely for toxicity while on therapy, and strategies for prevention and treatment of side effects should be further explored.

**Table 2:** Select ongoing clinical trials on immunotherapy in advanced HCC.

Clinical Trial	Phase	Intervention	Primary Endpoint(s)	Setting	Recruitment Status
RATIONALE-301 (NCT03412773) (59)	3	Tislelizumab, Sorafenib	OS, Safety	First-line	Active, not recruiting
CheckMate-9DW (NCT04039607) (60)	3	Nivolumab + Ipilimumab, Sorafenib, Lenvatinib	OS	First-line	Active, not recruiting
HIMALAYA (NCT03298451) (61)	3	Durvalumab + Tremelimumab, Durvalumab, Sorafenib	OS	First-line	Recruiting
RELATIVITY-073 (NCT04567615) (62)	2	Nivolumab + Relatlimab, Nivolumab	ORR	Second-line	Recruiting
NCT03680508 (63)	2	Cobolimab + Dostarlimab	ORR	First-line	Recruiting
LEAP-002 (NCT03713593) (64)	3	Lenvatinib + Pembrolizumab, Lenvatinib	PFS, OS	First-line	Active, not recruiting
COSMIC-312 (NCT03755791) (65)	3	Cabozantinib + Atezolizumab, Sorafenib, Cabozantinib	PFS, OS	First-line	Recruiting
NCT03764293 (66)	3	Apatinib + Camrelizumab, Sorafenib	PFS, OS	First-line	Recruiting
DEDUCTIVE (NCT03970616) (67)	1b/2	Tivozanib + Durvalumab	Safety	First and Second-line	Recruiting
NCT04183088 (68)	2	Regorafenib + Tislelizumab	Safety, ORR, PFS	First-line	Recruiting
GOING (NCT04170556) (69)	1/2	Regorafenib followed by Nivolumab	Safety	Second-line	Recruiting
RENOBATE (NCT04310709) (70)	2	Regorafenib + Nivolumab	ORR	First-line	Recruiting
REGSIN (NCT04718909) (71)	2	Regorafenib + Sintilimab	PFS	Second-line	Recruiting
ORIENT-32 (NCT03794440) (72)	2/3	Sintilimab + IBI305, Sorafenib	PFS, OS	First-line	Active, not recruiting
NCT05022927 (73)	1	ERY974 + Atezolizumab + Bevacizumab	Safety, ORR	Unspecified	Recruiting
NCT03198546 (74)	1	GPC3 and/or TGFβ targeting CAR-T cells	Dose-limiting toxicity	Unspecified	Recruiting
GLYCAR (NCT02905188) (75)	1	GLYCAR T cells + Cytoxan + Fludarabine	Dose-limiting toxicity	Unspecified	Recruiting

### ICI Plus Anti-Angiogenesis Combinations

After the success of the IMbrave-150 trial, additional combinations of ICI plus anti-angiogenesis therapy (TKIs, mAb) are currently under development (12). Angiogenesis plays an important role in modulating the tumor microenvironment through inhibition of APCs and effectors cells as well as activation of inhibitory cells including Tregs and TAMs. Thus, the addition of anti-angiogenic agents to ICIs has the potential to boost the anti-tumor immune response and provides the basis for combination studies (79). KEYNOTE-524 was a phase Ib study that demonstrated promising activity of lenvatinib plus pembrolizumab with 46% ORR by mRECIST, median OS of 22 months, and manageable toxicity (80). Based on these results, the ongoing phase III LEAP-002 trial (NCT03713593) is evaluating lenvatinib with or without pembrolizumab in the first-line setting for unresectable HCC (64). The phase III COSMIC-312 trial (NCT03755791) of cabozantinib plus atezolizumab was recently announced by press release to improve PFS compared to sorafenib in untreated HCC at primary analysis, although the OS endpoint was missed and final results are pending (65, 81). Other ICI/anti-angiogenesis combinations such as apatinib plus camrelizumab (NCT03764293), tivozanib plus durvalumab (NCT03970616), and regorafenib plus tiselizumab (NCT04183088), are under current investigation in clinical trials (66–68).

### Bispecific Antibodies

There are a variety of other immunotherapy-based approaches that are being evaluated in HCC besides ICIs. Bispecific antibodies are designed to recognize and bind specific tumor-associated antigens (e.g. AFP, GPC3) and effector cell receptors (e.g. CD3, CD28) in order to initiate tumor cytotoxicity (82). Ishiguro and colleagues successfully treated solid tumors in mouse models with a bispecific antibody (ERY974) combining CD3 on T cells and GPC3, which is expressed in most cases of HCC (83). Based on this preclinical data, a phase I study of ERY974 in combination with atezolizumab and bevacizumab in locally advanced or metastatic HCC is now ongoing (NCT05022927) (73). Other preclinical studies have demonstrated efficacy of bispecific antibodies targeting VEGF and EpCAM on HCC cancer cells in mice, providing basis for additional studies in humans (82).

### Cancer Vaccines and Oncolytic Viruses

Vaccine therapy is another strategy to help enhance the anti-tumor immune response by using artifically designed tumor antigens (based on nucleic acids, peptides, or dendritic cells) to prime cytotoxic T cells (78). AFP-based vaccines were used in earlier trials and have since expanded to other tumor antigens such as GPC3 and hTERT (78, 84). Although studies of vaccines in HCC have generally had limited success, combining them with ICIs and other agents warrants further examination (78). Oncolytic viruses are designed to preferentially replicate in cancer cells and result in tumor cell lysis, causing release of tumor antigens that activate anti-tumor immune responses (28). The modified poxvirus Pexa-Vec (JX-594) is the most widely studied oncolytic virus in HCC and has shown clinical activity as well as tolerability in previous studies (85). PHOCUS was a phase III clinical trial evaluating Pexa-Vec followed by sorafenib versus sorafenib in advanced HCC, however the study was discontinued after interim analysis indicated the unlikelihood of meeting the OS primary endpoint (86, 87).

### Adoptive Cell Therapy

Adoptive cell therapy is a method in which cells (e.g. NK cells, tumor-infiltrating lymphocytes, chimeric antigen receptor [CAR] T cells) are genetically engineered ex-vivo to target cancer cells and then reinfused into patients (88). Shi and colleagues discussed results from two phase I studies that enrolled 13 patients with advanced HCC treated with GPC3-CAR T cells; preliminary data showed 2 partial responses, 1-year OS rate of 42%, and most common toxicities included pyrexia, lymphopenia, and cytokine release syndrome (89). CAR T cell trials targeting other tumor antigens including EpCAM and TGF-β in addition to GPC3 are underway (74, 75, 90). We eagerly await the results from the aforementioned clinical trials.

## Conclusion

Over the past decade, the treatment paradigm for advanced HCC has dramatically changed. Molecular targeted agents represented the first important advancement in HCC treatment after a long history of ineffective cytotoxic chemotherapy. Despite this progress, long-term survival of patients with advanced stage disease remains low, highlighting the need for further improvement of therapy. In recent years, immunotherapy has changed the landscape of cancer treatment. Interest in its application for HCC has significantly grown given long lasting benefits seen in a small subset of patients, which propelled several studies evaluating other ICI combinations and novel modalities of immunotherapy. Further research will be needed to identify biomarkers to determine which patients will benefit from immunotherapy and establish the safety and efficacy in those with poor liver function, especially given their very limited treatment options. With more patients receiving ICI-based frontline therapy, future studies will also need to determine sequencing of treatments after immunotherapy exposure and investigate managing immunotherapy resistance and toxicity.

## Author Contributions

SK, LK, and MA: study conception, design, and drafting of manuscript. SK and LK: acquisition of data. SK and MA: analysis and interpretation of data. SK, LK, BFE-R, and MA: critical revision. All authors contributed to the article and approved the submitted version.

## Conflict of Interest

The authors declare that the research was conducted in the absence of any commercial or financial relationships that could be construed as a potential conflict of interest.

## Publisher’s Note

All claims expressed in this article are solely those of the authors and do not necessarily represent those of their affiliated organizations, or those of the publisher, the editors and the reviewers. Any product that may be evaluated in this article, or claim that may be made by its manufacturer, is not guaranteed or endorsed by the publisher.
